# Assessment of productivity, nutrient uptake and economic benefits of rice under different nitrogen management strategies

**DOI:** 10.7717/peerj.9596

**Published:** 2020-07-29

**Authors:** Guoying Yang, Hongting Ji, Hongjiang Liu, Yuefang Zhang, Liugen Chen, Jianchu Zheng, Zhi Guo, Jing Sheng

**Affiliations:** 1 Circular Agriculture Research Center, Jiangsu Academy of Agricultural Sciences, Nanjing, Jiangsu, China; 2 Key Laboratory for Crop and Animal Integrated Farming, Ministry of Agriculture, Nanjing, Jiangsu, China; 3 Nanjing Institute of Agricultural Sciences in Jiangsu Hilly Area, Jiangsu Academy of Agricultural Sciences, Nanjing, Jiangsu, China

**Keywords:** Organic–inorganic compound fertilizer, Crop productivity, Nutrient use efficiency, Economic benefit, Principal component analysis, Sustainability, Slow-release fertilizer

## Abstract

**Background:**

Integrating a chemical nitrogen (N) fertilizer with an organic fertilizer and using slow-release mechanism are important N management strategies to increase the N utilization efficiency (NUE) and grain yield of rice. However, the performances of both N management strategies on the productivity, the nutrient absorption and utilization efficiency, and the economic benefits of rice have not yet been comprehensively evaluated.

**Methods:**

A 2-year field experiment was conducted with seven N management strategies without fertilizer (control), 100% conventional N fertilizer (conventional compound fertilizer and urea) (N_100_), 75% conventional N fertilizer with 25% organic–inorganic compound fertilizer (N_75_+OICF_25_), 50% conventional N fertilizer with 50% organic–inorganic compound fertilizer (N_50_+OICF_50_), 100% organic–inorganic compound fertilizer (OICF_100_), slow-release compound fertilizer with urea (SRCF+U), compound fertilizer with sulfur-coated urea (CF+SCU). The responses of the productivity, the nutrient absorption and utilization efficiency, and the economic benefits of rice to the different N management strategies were evaluated.

**Results:**

CF+SCU performed comparably or better than N_100_, judging by the grain yield (GY), the N, phosphate (P) and potassium (K) agronomic efficiency (NAE, PAE and KAE), and the apparent N, P and K recovery efficiency (ANRE, APRE and AKRE). SRCF+U significantly increased the GY by an average of 7.7%, the NAE and the ANRE by 23.8 and 26.7%, the PAE and the APRE by 90.6 and 109.3%, and the KAE and the AKRE by 74.2 and 57.7%. The higher GY and nutrient utilization efficiency when using SRCF+U were attributed to the higher total biomass and total nutrient absorption. N_75_+OICF_25_ and N_50_+OICF_50_ produced a comparable grain yield than N_100_, whereas a significant yield reduction was observed when using OICF_100_. Compared with N_100_, N_75_+OICF_25_ resulted in a comparable or higher fertilizer use efficiency (0.3 and 4.7% for NAE and ANRE, 0.3 and 3.2% for PAE and APRE, 0.3 and −2.8% for KAE and AKRE). However, the fertilizer use efficiency when using N_50_+OICF_50_ and OICF_100_ were lower than with N_100_. The highest net return (NR) (5,845.03 yuan ha^−1^) and benefit to cost (B:C) ratio (0.34) were obtained when using SRCF+U. The NR and the B:C ratio when using N_75_+OICF_25_ were slightly higher than when using N_100._ However, N_50_+OICF_50_ and OICF_100_ significantly decreased the NR and the B:C ratio compared with N_100_ by 14.5 and 12.1% and by 35.1 and 29.0%, respectively.

**Conclusions:**

SRCF+U and CF+SCU enhanced the crop productivity, the nutrient uptake and utilization efficiency, and the economic benefits compared with N_100._ The comprehensive performance of SRCF+U was better than that of CF+SCU. N_75_+OICF_25_ produced almost similar productivity, nutrient uptake and use efficiency compared with N_100_. It demonstrated that N_75_+OICF_25_ stabilized the grain yield production of rice and reduced the input of chemical N fertilizer.

## Introduction

Rice is one of the major food crops in the world. In China, rice accounts for one-third of the total planting area and is a staple food for more than 60% of the population (Guo et al., 2015). China is also the largest consumer of chemical nitrogen (N) fertilizer worldwide and is responsible for 37% of the global N fertilizer consumption (Peng et al., 2011). However, the current N utilization efficiency (NUE) of rice is only 20–30% (Peng et al., 2006). The low NUE is due to excessive and improper N use and triggers several environmental problems, including water eutrophication, soil acidification and ammonia volatilization (Ju et al., 2009; Spiertz, 2010; Peng et al., 2011).

To increase the economic benefits for the farmers and reduce the environmental pollution, many N saving application patterns have been developed. Integrating the chemical N fertilizer with the organic N fertilizer has been suggested as one of the most effective methods to stabilize rice production, while decreasing the N input (Wei et al., 2016; Mi et al., 2018; Singh et al., 2019). Many previous studies showed that the combined use of chemical N fertilizer with organic N fertilizer increased the NUE and the rice grain yield and improved the soil fertility (Xu et al., 2008; Meng et al., 2009; Lv et al., 2017; Yang et al., 2019). Recently, organic–inorganic compound fertilizer (OICF) was introduced as a new type of fertilizer in China (Zhao et al., 2012). Compared with the conventional organic fertilizer, the OICF resulted in a higher fertilizer use efficiency, a lower labor cost, and less environmental pollution (Ni et al., 2010). However, there were very few studies on the fertilization strategy for this new fertilizer in rice production (Li et al., 2015). Previous researches have studied the impact of a single application of OICF on the rice yield, NUE and soil fertility (Zhang, Xu & Zhang, 2009; Tian et al., 2012). However, a single use of the OICF was not enough to meet the nutritious requirement of the rice plants in different growth stages because of the slow N mineralization from organic materials (Wei et al., 2016; Iqbal et al., 2019). The combined use of OICF and conventional chemical N fertilizer could enhance the availability of soil nutrients, the physical condition of the soil, and improve the microbial community diversity and enzyme activity of the soil (Lu et al., 2015; Zhao et al., 2016), thereby promoting the N absorption and utilization as well as the grain yield (Abbasi & Tahir, 2012; Zhao et al., 2016; Li et al., 2017). However, there is only limited information available on the proportions of OICF and conventional chemical N fertilizer to be used. Zhao et al. (2016) reported the impact of using an OICF with a chemical N fertilizer on the rice yield. They showed that using 70% OICF as the basal fertilizer with 30% chemical N fertilizer as panicle fertilizer increased the rice yield compared with using chemical fertilizer alone. Li et al. (2015) reported that using 55% OICF as basal fertilizer with 45% chemical N fertilizer as the panicle fertilizer increased the rice yield by 2.3% and the gross return by 2.3% compared with using 100% chemical N fertilizer.

Similar to organic fertilizers, slow-release fertilizers are designed to release the fertilizer into the soil at a rate that meets the nutritious requirement of the rice plants compared with conventional chemical fertilizers (Wang et al., 2011, 2018; Zheng et al., 2016). Therefore, it could save chemical fertilizer and enhance nutrient utilization efficiency. However, slow-release fertilizers are generally too expensive to use in crop production in comparison with conventional chemical fertilizers (Ni et al., 2010). The combined use of a slow-release fertilizer with a conventional fertilizer improved the NUE and the rice grain yield while decreasing the cost and the labor required (Ye et al., 2013; Zheng et al., 2016).

Although many previous studies have reported an increase of the grain yield, the NUE and the economic benefits of rice individually under the combined application of a chemical N fertilizer and an OICF or a slow-release fertilizer, the comprehensive assessment of the impact of these N management strategies on the crop productivity, the nutrient absorption and utilization efficiency, and the economic benefits of rice have not been realized. Therefore, this study aimed at assessing the performances of the different N fertilizer management strategies for rice production. The performance was assessed by analyzing the effects of the different N fertilizer management strategies on the grain yield, the total biomass, the harvest index, the total nutrient absorption, the grain nutrient absorption, the nutrient harvest index, the nutrient utilization efficiency, and the economic benefits. A secondary goal is to comprehensively evaluate the performance of the different N fertilizer management strategies based on the principal component analysis. Our study provides a useful basis for the management of N fertilizer by rice growers.

## Materials and Methods

### Experimental site

A 2-year experiment was conducted in a paddy field under a rice-wheat rotation in Yangzhong, Zhenjiang, Jiangsu, China (32°12′ N, 119°85′ E) during the 2018 and 2019 rice growing seasons. The use of paddy field in which we conduct the experiment was authorized by Jiangsu Zijiang Ecological Agriculture Co., Ltd. The soil was classified as Hydragric Anthrosolos. The initial properties of the soil are presented in Table 1.

**Table 1:** The initial properties of the soil in the study.

Soil properties	Values	Method
pH	6.38	Measured by a portable pH meter.
Organic matter	22.80 g kg^−1^	Potassium dichromate volumetric method (Bao, 2000).
Total nitrogen (N)	0.98 g kg^−1^	Kjeldahl digestion method (Nelson & Somers, 1973)
Total phosphorus (P)	0.57 g kg^−1^	Molybdovanadate method (Soon & Kalra, 1995)
Available N	143.1 mg kg^−1^	Diffusion method (Bao, 2000)
Available P	10.95 mg P_2_0_5_ kg^−1^	Olsen’s method (Black, 1965)
Available K	98.64 mg K_2_O kg^−1^	Flame photometry (Walker & Barker, 1962)

### Experimental design

The experiments were performed in a completely randomized block with three replicates, and the area of experimental plot in each treatment was about 180 m^2^ (length × width = 20 m × 9 m). The different treatments were: a control without any chemical fertilizer (control), 100% conventional N fertilizer (conventional compound fertilizer and urea) (N_100_), 75% conventional N fertilizer with 25% organic–inorganic compound fertilizer (N_75_+OICF_25_), 50% conventional N fertilizer with 50% organic–inorganic compound fertilizer (N_50_+OICF_50_), 100% organic–inorganic compound fertilizer (OICF_100_), slow-release compound fertilizer with urea (SRCF+U), and compound fertilizer with sulfur-coated urea (CF+SCU). The description of the fertilizers application in this study is given in Table 2.

**Table 2:** Description of fertilizer application in the study.

Treatment	N (kg ha^-1^)	P_2_O_5_ (kg ha^-1^)	K_2_O (kg ha^-1^)	Basal fertilizer (kg ha^-1^)	Tiller fertilizer (kg ha^-1^)	Panicle fertilizer (kg ha^-1^)
Control	–	–	–	–	–	–
N_100_	273.75	101.3	101.3	375 conventional compound fertilizer (N-P_2_O_5_-K_2_O = 15%-15%-15%) + 150 urea (N = 46%)	150 urea (N = 46%)	300 conventional compound fertilizer (N-P_2_O_5_-K_2_O = 15%-15%-15%) + 75 urea (N = 46%)
N_75_+OICF_25_	273.75	101.3	101.3	977.7 organic–inorganic compound fertilizer (N-P-K =7%-4%-4%) + 123.9 urea (N = 46%)	150 urea (N = 46%)	414.3 conventional compound fertilizer (N-P_2_O_5_-K_2_O = 15%-15%-15%) + 37.5 (urea, N = 46%)
N_50_+OICF_50_	273.75	101.3	101.3	1955.4 organic–inorganic compound fertilizer (N-P-K =7%-4%-4%)	124.7 (urea, N = 46%)	153.6 conventional compound fertilizer (N-P_2_O_5_-K_2_O = 15%-15%-15%) + 122.7 (urea, N = 46%)
OICF_100_	273.75	156.4	156.4	3910.7 organic–inorganic compound fertilizer (N-P-K =7%-4%-4%)	–	–
SRCF+U	273.75	65.5	78.6	482 slow-release compound fertilizer (N-P-K = 26%-10%-12%)	150 (urea, N = 46%)	173 slow-release compound fertilizer (N-P_2_O_5_-K_2_O = 26%-10%-12%) + 75 (urea, N = 46%)
CF+SCU	273.75	101.3	101.3	375 conventional compound fertilizer (N-P_2_O_5_-K_2_O =15%-15%-15%) +375 sulfur-coated urea (N = 37%)	–	300 compound fertilizer (N-P_2_O_5_-K_2_O = 15%-15%-15%) + 93 sulfur-coated urea (N = 37%)

The amount of N fertilizer was 273.75 kg ha^−1^ under each fertilized treatment. A total of 70% of the N fertilizer was used as basal-tillering fertilizer, and 30% was used as panicle fertilizer. The conventional N application scheme in rice production (N_100_) was followed: 375 kg ha^−1^ conventional compound fertilizer (N-P_2_O_5_-K_2_O = 15%-15%-15%) and 150 kg ha^−1^ urea (N = 46%) as basal fertilizer, 150 kg ha^−1^ urea as tillering fertilizer, 300 kg ha^−1^ conventional compound fertilizer and 75 kg ha^−1^ urea as panicle fertilizer. Generally, organic–organic compound fertilizers are used as basal fertilizer in rice production (Li et al., 2015; Zhao et al., 2016). Thus, the organic–inorganic compound fertilizer (N-P_2_O_5_-K_2_O = 7%-4%-4%, organic matter ≥ 20%, moisture content ≤ 12%) replaced the chemical fertilizer during the basal fertilization period. Both the slow-release compound fertilizer (N-P_2_O_5_-K_2_O = 26%-10%-12%, the initial release rate of nutrient ≤15%, the 28-day cumulative release rate of nutrient ≤80%, the stated release time of 90–120 d) and the sulfur-coated urea (N = 37%, the initial release rate of nutrient ≤25%, the 28-day cumulative release rate of nutrient ≤80%, the stated release time of 60–90 d) replaced the chemical fertilizer during the basal stage and the panicle stage. The slow-release compound fertilizer was used to replace the conventional compound fertilizer, and the sulfur-coated urea was used to replace urea. The rates of phosphorus (P) and potassium (K) fertilizer were 156.4 and 156.4 kg ha^−1^ for OICF_100_, and 65.5 and 78.6 kg ha^−1^ for SRCF+U, respectively. The rates of P and K fertilizer for the other fertilized treatments were 101.3 and 101.3 kg ha^−1^, respectively. Since the N, P, and K in the OICF is fixed, the rates of P and K fertilizer under OICF_100_ increase for the same N rate than in N_100_. The use of slow-release compound fertilizer in the SRCF+U treatment led to the reduction of the P and K use compared with the N_100_ treatment. The experimental cultivar was Nanjing 2,728, a medium-maturing medium japonica rice cultivar, currently used in the local production. A mechanical rice transplanter (YANMAR VPE 6, Japan) was used to transplant the rice seedlings into the plots with a density of 2 plants hill^−1^. The hill spacing was 30 cm × 14 cm. All other cultivation practices were conducted in line with the rice cultivation standards of high-yield rice.

### Measurement and analysis

At least ten hills of rice plants in each plot were sampled at maturity. The samples were first killed out at 105 °C for 30 min, then dried at 80 °C until a constant weight was reached to measure the dry matter weight. The parts of the plants were sieved with a 1-mm sieve after grinding through a grinder machine. The concentration of N, P and K were determined after wet digestion with H_2_SO_4_ and H_2_O_2_. The concentration of N was determined by the Kjeldahl digestion method (Nelson & Somers, 1973), the concentration of P by the molybdovanadate method (Soon & Kalra, 1995), and the concentration of K by the flame photometry (Walker & Barker, 1962). At maturity, 2 m^2^ of rice plants with three replicates (6 m^2^ of rice plants in total) were sampled in each plot to determine the actual grain yield. The grain yield was modified to a moisture content of 14% of the fresh weight.

### Use efficiency of N, P and K

The N, P and K agronomic efficiency (*NAE*, *PAE* and *KAE*) and the apparent N, P and K recovery efficiency (A*NRE*, A*PRE* and A*KRE*) were calculated according to the following equations:

$${\rm NAE\; (\% )\; = \; }\displaystyle{{{Y - }{{Y}_{\rm 0}}} \over {{{F}_{\rm N}}}}{\rm \; \times \; 100}$$

$${\rm PAE\ (\% )\ = \ }\displaystyle{{{Y - }{{Y}_{\rm 0}}} \over {{{F}_{\rm P}}}}{\rm\ \times\ 100}$$

$${\rm KAE \ (\% )\ = \ }\displaystyle{{{Y - }{{Y}_{\rm 0}}} \over {{{F}_{\rm K}}}}{\rm \ \times\ 100}$$

$${\rm ANRE\ (\% )\ = }\displaystyle{{{N - }{{N}_{\rm 0}}} \over {{{F}_{\rm N}}}}{\rm\ \times\ 100}$$

$${\rm APRE\ (\% )\ = \ }\displaystyle{{{P - }{{P}_{\rm 0}}} \over {{{F}_{\rm P}}}}{\ \times\ 100}$$

$${\rm AKRE\ (\% )\ = \ }\displaystyle{{{K - }{{K}_{\rm 0}}} \over {{{F}_{\rm K}}}}{\ \times\ 100}$$

*Y* and *Y*_0_ are the rice yield for fertilized treatment and the control, respectively. *F*_N_, *F*_P_ and *F*_K_ are the total N, total P and total K used, respectively. *N* and *N*_0_ are the total N uptake of rice plants for fertilized treatment and the control, respectively. *P* and *P*_0_ are the total P uptake of rice plants for fertilized treatment and the control, respectively. *K* and *K*_0_ are the total K uptake of rice plants for fertilized treatment and the control, respectively.

### Economic analysis

The cost of cultivation (yuan ha^−1^, 1 USD = 7.09 yuan) was calculated by adding the costs for all the inputs (namely the seed, nursery tray, substrate, agricultural film, irrigation, labor, fertilizer and chemicals, mechanical operation, grain drying, etc.). The average grain price of rice in 2 years was 2.6 yuan kg^−1^. The gross return (GR), net return (NR) and benefit to cost ratio (B:C ratio) were calculated as followed:

GR (yuan ha^−1^) = Grain yield × grain price

NR (yuan ha^−1^) = GR − Cost

B:C ratio = NR/Cost

### Statistical analysis

All the statistical analyses were carried out in SPSS 20.0. The impacts of the year and the N management strategy on the grain yield, the total biomass, the harvest index, the nutrient absorption and utilization efficiency, the NR and the B:C ratio were analyzed using a mixed linear model at *p* < 0.05. The year and the N management strategy were used as fixed factors, and replicate was used as a random factor. For each year, the effect of the different N management strategies on the grain yield, the total biomass, the harvest index, the nutrient uptake and utilization efficiency were tested using ANOVA. The differences between means were determined using the least significant difference test at *p* < 0.05. A principal component analysis was applied to investigate the impacts of the different N management strategies on the yield formation, the nutrient uptake and utilization efficiency and the economic benefits.

## Results

### Grain yield, total biomass and harvest index

**Figure 1:**
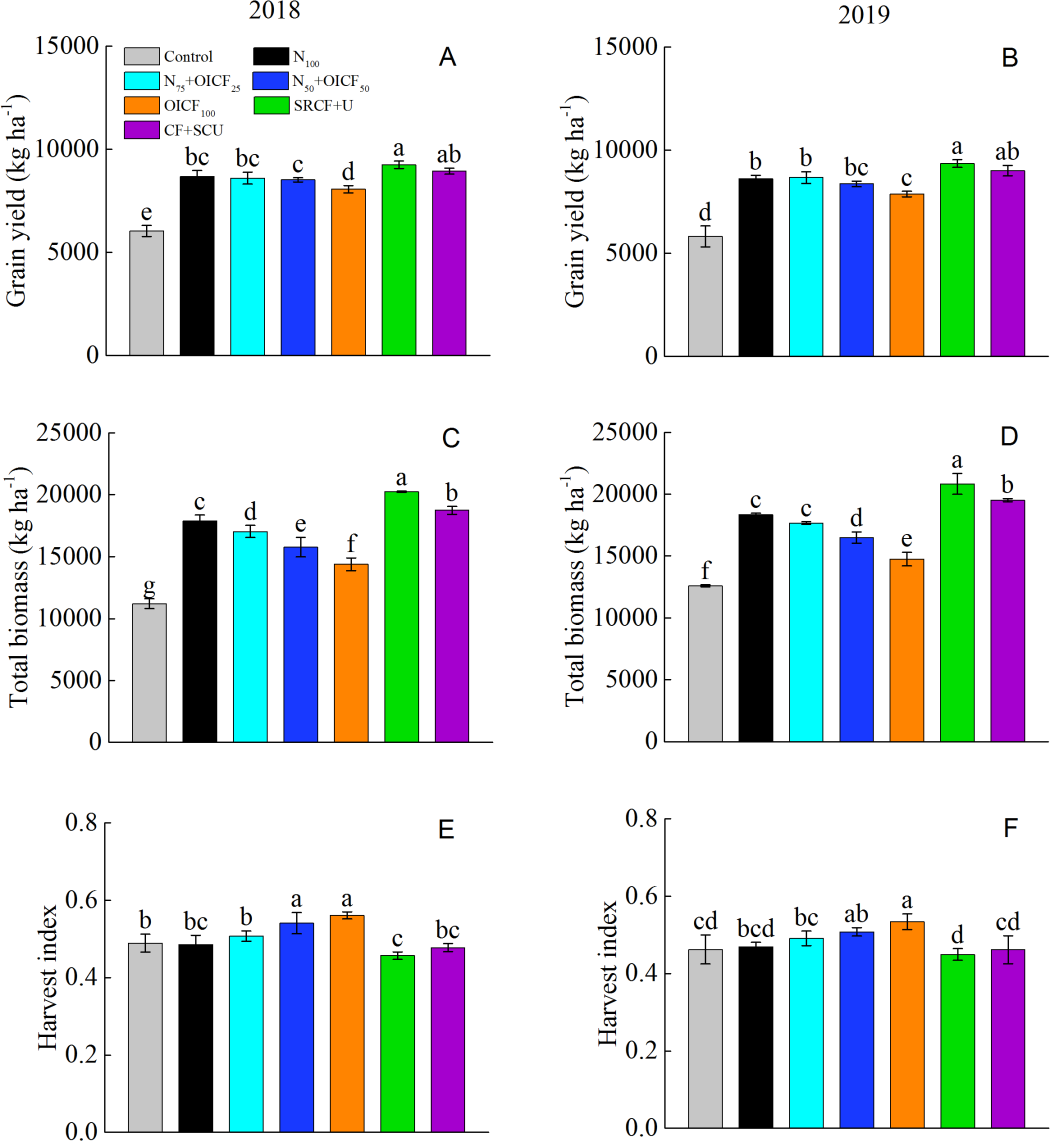
The grain yield (A and B), total biomass (C and D) and harvest index (E and F) of rice under the different nitrogen fertilizer managements during the 2018 and 2019 rice growing seasons. N_100_, 100% conventional nitrogen (N) fertilizer (conventional compound fertilizer and urea); N_75_+OICF_25_, 75% conventional N fertilizer with 25% organic–inorganic compound fertilizer; N_50_+OICF_50_, 50% conventional N fertilizer with 50% organic–inorganic compound fertilizer; OICF_100_, 100% organic–inorganic compound fertilizer; SRCF+U, slow-release compound fertilizer with urea; CF+SCU, compound fertilizer with sulfur-coated urea. Vertical bars indicate the standard deviation of mean (*n* = 3). Means within the same column followed by different letters are significantly different at the 0.05 level.

Table 3 shows that the effect of the year (Y) on the grain yield (GY) was not significant, whereas the Y had a significant influence on the total biomass (TB) and harvest index (HI) at the 0.05 level. The effect of the N management strategy (NMS) on the GY, TB and HI were significant (Table 3). Generally, the GY of rice was significantly higher when using the fertilized treatments than for the control. The grain yield when using the SRCF+U treatment significantly increased by 6.6–8.7% compared with N_100_. The CF+SCU, N_75_+OICF_25_ and N_50_+OICF_50_ treatments produced comparable yields than N_100_ treatment, whereas the yield significantly decreased by 7.1–8.6% when using the OICF_100_ (Figs. 1A and 1B). Similarly, the highest TB was obtained with the SRCF+U treatment, followed by the CF+SCU and the N_100_ treatments. The TB for N_75_+OICF_25_ slightly decreased compared with N_100_, whereas a significantly lower TB was obtained with the N_50_+OICF_50_ and OICF_100_ treatments (Figs. 1C and 1D). The maximum HI was obtained with the N_50_+OICF_50_ and OICF_100_ treatments, which indicated that the biomass was more efficiently allocated to the grain because of OICF application. The minimum HI was obtained for the SRCF+U and CF+SCU treatments, which were the highest yield-producing treatments (Figs. 1E and 1F). This indicated that the grain yield when using slow-release fertilizers (SRCF+U and CF+SCU) was more dependent on the TB than on the HI (Table 4).

**Table 3:** Probability values of the effects of year (Y), nitrogen management strategy (NMS) and their interaction (Y × NMS) on the grain yield, total biomass, harvest index, nutrient uptake and utilization efficiency, net return and benefit to cost ratio.

Growth index	Year (Y)	Nitrogen management strategies (NMS)	Y × NMS
Grain yield	0.59	<0.001	0.98
Total biomass	<0.001	<0.001	0.98
Harvest index	0.003	<0.001	0.93
Grain nitrogen (N) uptake	<0.001	<0.001	0.11
Total N uptake	0.22	<0.001	0.02
Grain phosphorus (P) uptake	0.27	<0.001	0.90
Total P uptake	0.13	<0.001	0.08
Grain potassium (K) uptake	0.56	<0.001	0.99
Total K uptake	<0.001	<0.001	<0.001
N harvest index	<0.001	<0.001	0.04
P harvest index	0.009	<0.001	0.15
K harvest index	0.16	<0.001	<0.001
N agronomic efficiency	0.54	<0.001	0.98
P agronomic efficiency	0.45	<0.001	0.43
K agronomic efficiency	0.45	<0.001	0.43
Apparent N recovery efficiency	<0.001	<0.001	0.26
Apparent P recovery efficiency	0.001	<0.001	0.008
Apparent K recovery efficiency	<0.001	<0.001	<0.001
Net return	0.48	<0.001	0.84
Benefit to cost ratio	0.49	<0.001	0.90

**Table 4:** Pearson correlation coefficients among the characteristics that were measured in this study.

Index	GY	TB	HI	GN	TN	GP	TP	GK	TK	NHI	PHI	KHI	NAE	PAE	KAE	ANRE	APRE	AKRE
Grain yield (GY)	1.00																	
Total biomass(TB)	0.91**	1.00																
Harvest index (HI)	−0.19	−0.56	1.00															
Grain nitrogen uptake (GN)	0.95**	0.77*	0.05	1.00														
Total N uptake (TN)	0.95**	0.96**	−0.42	0.88**	1.00													
Grain phosphorus uptake (GP)	0.97**	0.83*	−0.04	0.97**	0.90**	1.00												
Total P uptake (TP)	0.97**	0.97**	−0.41	0.88**	0.98**	0.91**	1.00											
Grain potassium uptake (GK)	0.97**	0.87**	−0.15	0.96**	0.94**	0.97**	0.95**	1.00										
Total K uptake (TK)	0.89**	0.99**	−0.61	0.75*	0.96**	0.80*	0.97**	0.87*	1.00									
N harvest index (NHI)	−0.68	-0.88**	0.80*	−0.51	-0.85*	−0.57	−0.81*	−0.66	−0.92**	1.00								
P harvest index (PHI)	−0.45	−0.74	0.91**	−0.25	−0.64	−0.28	−0.64	−0.41	−0.78*	0.89**	1.00							
K harvest index (KHI)	−0.10	−0.47	0.97**	0.12	−0.34	0.07	−0.31	−0.04	−0.51	0.76*	0.88**	1.00						
N agronomic efficiency (NAE)	1.00**	0.91**	−0.19	0.95**	0.95**	0.97**	0.97**	0.97**	0.89**	−0.68	−0.45	−0.10	1.00					
P agronomic efficiency (PAE)	0.93**	0.95**	−0.43	0.82*	0.93**	0.85*	0.96**	0.88**	0.93**	−0.75	−0.66	−0.33	0.93**	1.00				
K agronomic efficiency (KAE)	0.91**	0.96**	−0.49	0.81*	0.94**	0.83*	0.96**	0.87*	0.95**	−0.80*	−0.71	−0.40	0.91**	0.99**	1.00			
Apparent N recovery efficiency (ANRE)	0.95**	0.96**	−0.42	0.88**	1.00**	0.90**	0.98**	0.94**	0.96**	−0.85*	−0.64	−0.34	0.95**	0.93**	0.94**	1.00		
Apparent P recovery efficiency (APRE)	0.85*	0.94**	−0.57	0.72	0.88**	0.75	0.92**	0.80*	0.93**	−0.79*	−0.76*	−0.46	0.85*	0.98**	0.98**	0.88**	1.00	
Apparent K recovery efficiency (AKRE)	0.85*	0.97**	−0.63	0.72	0.93**	0.75	0.95**	0.82*	0.97**	−0.88**	−0.82*	−0.54	0.85*	0.96**	0.98**	0.93**	0.98**	1.00

**Notes:**

* *p* < 0.05.

** *p* < 0.01.

### N, P and K uptake

**Figure 2:**
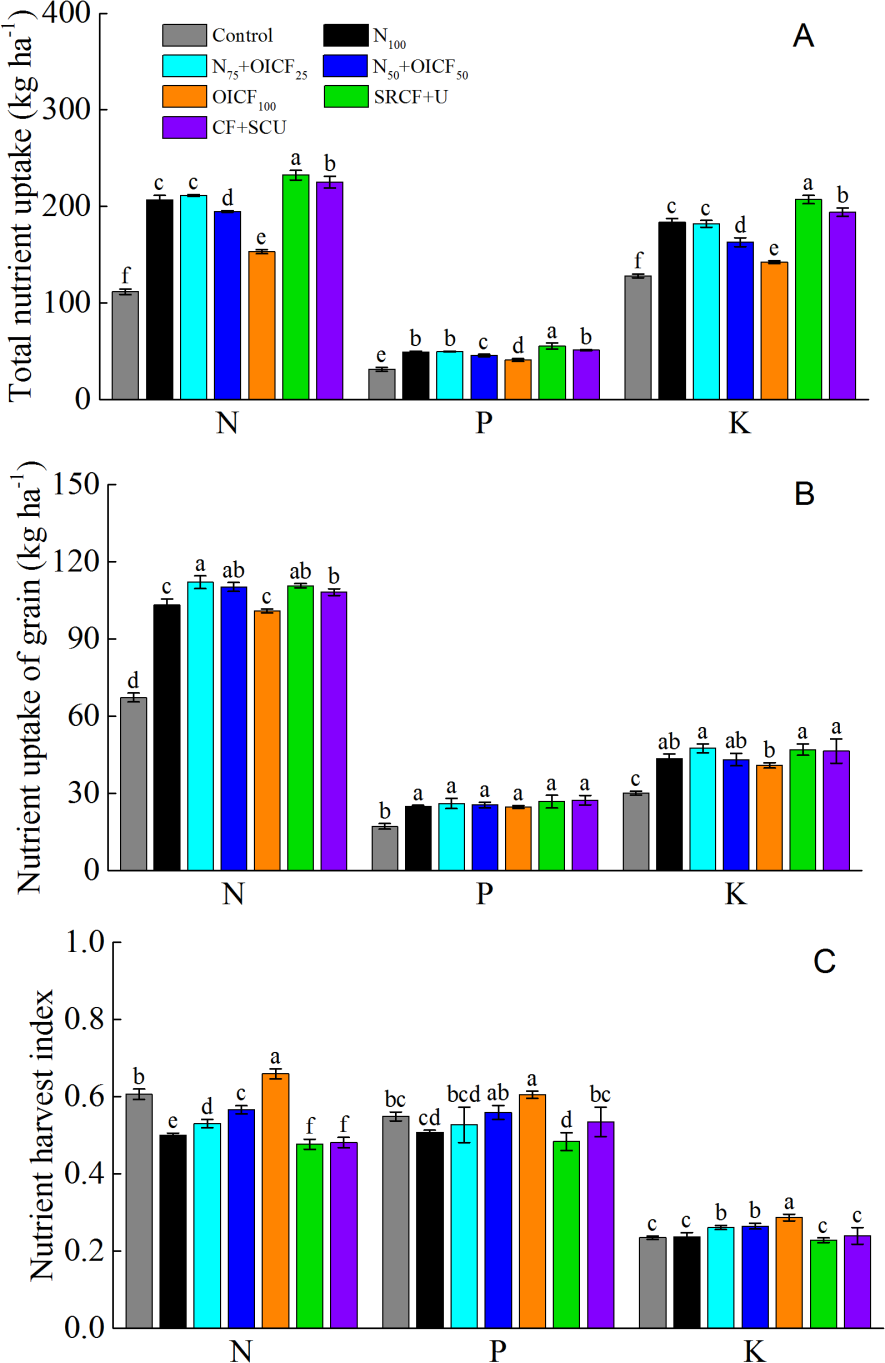
The total nutrient uptake (A), grain nutrient uptake (B) and nutrient harvest index (C) of rice under the different nitrogen fertilizer management strategies. N_100_, 100% conventional nitrogen (N) fertilizer (conventional compound fertilizer and urea); N_75_+OICF_25_, 75% conventional N fertilizer with 25% organic–inorganic compound fertilizer; N_50_+OICF_50_, 50% conventional N fertilizer with 50% organic–inorganic compound fertilizer; OICF_100_, 100% organic–inorganic compound fertilizer; SRCF+U, slow-release compound fertilizer with urea; CF+SCU, compound fertilizer with sulfur-coated urea. Vertical bars indicate the standard deviation of mean (*n* = 3). Means within the same column followed by different letters are significantly different at the 0.05 level.

Figure 2 shows the nutrient uptake for different N management strategies. The fertilized treatments significantly increased the nutrient uptake compared with the control. The total N uptake (TN) was markedly higher for SRCF+U and CF+SCU treatments compared to N_100_, N_75_+OICF_25_, N_50_+OICF_50_ and OICF_100_ treatments. There was no difference in TN for the N_75_+OICF_25_ compared to the N_100_ treatment, whereas the TN was significantly lower for the N_50_+OICF_50_ and OICF_100_ treatments compared to the N_100_ treatment. The highest grain N uptake (GN) was observed for the N_75_+OICF_25_, followed by N_50_+OICF_50_ and SRCF+U. The GN for the OICF_100_ treatment was significantly lower than for N_75_+OICF_25_ and N_50_+OICF_50_. No significant difference was observed between N_100_ and OICF_100_ or between SRCF+U and CF+SCU. The highest N harvest index (NHI) was obtained for the OICF_100_ treatment, followed by N_50_+OICF_50_, N_75_+OICF_25_, N_100_. The lowest value was obtained for SRCF+U (Fig. 2).

The total P uptake (TP) was the highest for SRCF+U and was dramatically higher for the other treatments. No significant difference in the TP was observed for N_100_, N_75_+OICF_25_ and CF+SCU. A significantly lower TP was measured for the N_50_+OICF_50_ and OICF_100_ compared with N_100_. There was insignificant difference in the grain P uptake (GP) between the fertilized treatments. The highest P harvest index (PHI) was observed for OICF_100_, followed by N_50_+OICF_50_ and the control. The lowest PHI was obtained for SRCF+U (Fig. 2).

Similar to the TP, the highest total K uptake (TK) was obtained for SRCF+U, followed by CF+SCU. The lowest was observed for the control. We did not observe any significant difference in the TK between N_100_ and N_75_+OICF_25_. But the TK values for the N_50_+OICF_50_ and OICF_100_ were significantly lower than for N_100_. The highest grain K uptake (GK) was observed for the SRCF+U, followed by N_75_+OICF_25_, CF+SCU, N_100_, N_50_+OICF_50_ and OICF_100_. The minimum value was obtained for the control. The highest K harvest index (KHI) was obtained for OICF_100_, followed by N_50_+OICF_50_ and N_75_+OICF_25_, whereas the lowest value was detected for SRCF+U. There was no difference among the N_100_, SRCF+U and CF+SCU treatments (Fig. 2).

In general, the effect of the Y on TN, GP, TP, GK and KHI was not significant, but GN, TK, NHI and PHI strongly depended on it (*p* < 0.05). As expected, the effect of the NMS on TN, TP, TK, GN, GP, GK, NHI, PHI and KHI was significant at the 0.05 level. Moreover, the interaction effects of Y and NMS on GN, GP, TP, GK and PHI were not significant, whereas they were significant for TN, TK, NHI and KHI (*p* < 0.01) (Table 3).

A simple correlation analysis showed that the GN, the GP and the GK were significantly positively correlated with the TN, the TP and the TK, respectively. In contrast, the GN, the GP and the GK were negatively correlated with the NHI, the PHI and the KHI but not significantly. Significantly positive correlations were observed among GN, GP and GK or among NHI, PHI and KHI (Table 4).

### Nutrient utilization efficiency

**Figure 3:**
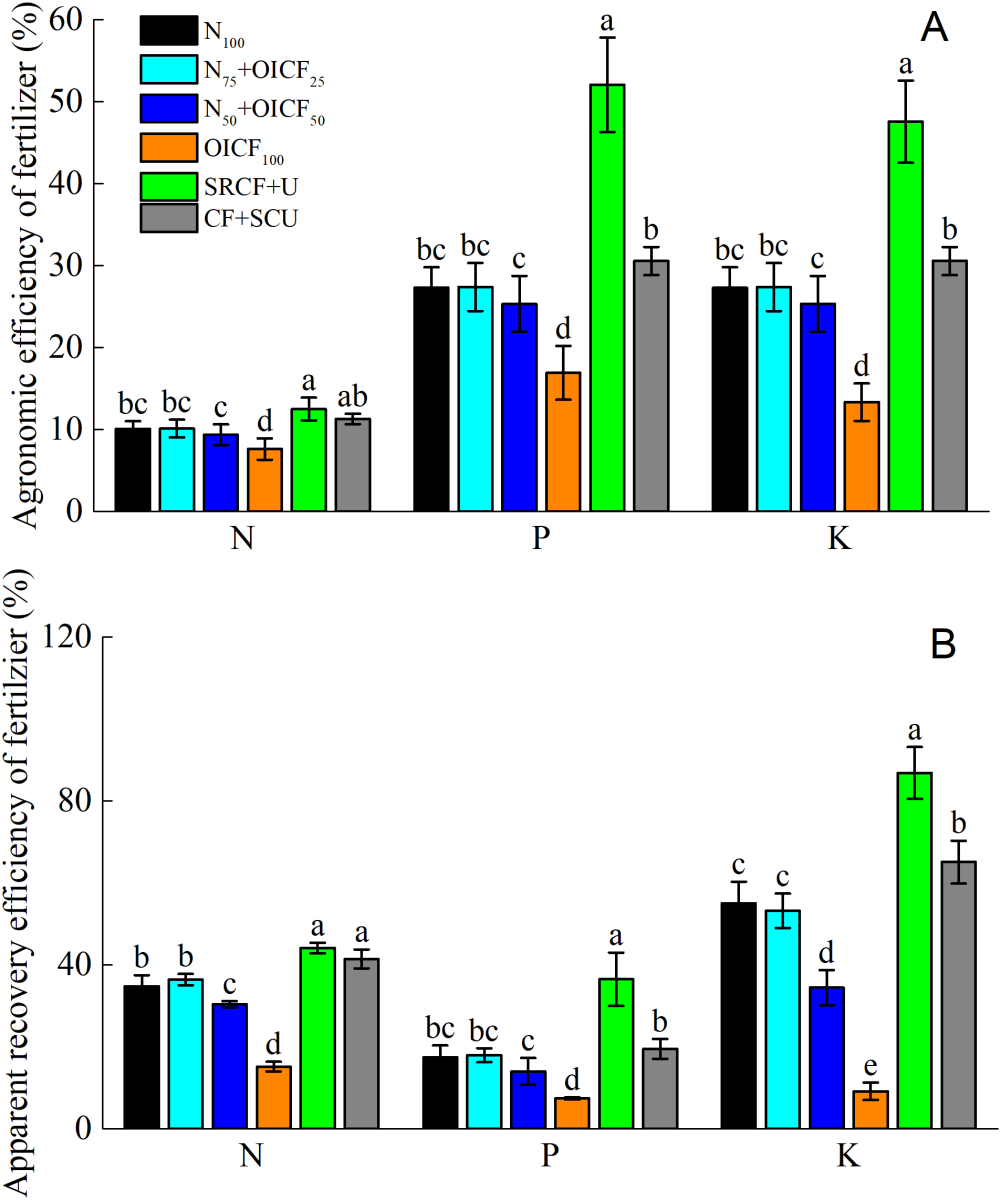
The agronomic efficiency (A) and apparent recovery efficiency (B) of fertilizer under the different nitrogen fertilizer management strategies. N_100_, 100% conventional nitrogen (N) fertilizer (conventional compound fertilizer and urea); N_75_+OICF_25_, 75% conventional N fertilizer with 25% organic–inorganic compound fertilizer; N_50_+OICF_50_, 50% conventional N fertilizer with 50% organic–inorganic compound fertilizer; OICF_100_, 100% organic–inorganic compound fertilizer; SRCF+U, slow-release compound fertilizer with urea; CF+SCU, compound fertilizer with sulfur-coated urea. Vertical bars indicate the standard deviation of mean (*n* = 3). Means within the same column followed by different letters are significantly different at the 0.05 level.

Figure 3 shows the fertilizer agronomic efficiency and the apparent fertilizer recovery efficiency under the different N management strategies. The highest N, P and K agronomic efficiency (NAE, PAE and KAE) and the highest apparent N, P and K recovery efficiency (ANRE, APRE and AKRE) were observed for SRCF+U, followed by CF+SCU, whereas the lowest values were obtained for OICF_100_. There was no significant difference in the NAE and ANRE between SRCF+U and CF+SCU. However, the PAE, the KAE, the APRE, and the AKRE under SRCF+U were significantly higher than those for CF+SCU. This indicated that the application of SRCF significantly increased P and K use efficiency compared with SCU. Compared with N_100_, there was insignificant difference in the NAE, the PAE and the KAE for N_75_+OICF_25_ and N_50_+OICF_50_. On the contrary, the NAE, the PAE and the KAE for OICF_100_ were significantly lower than for the other fertilization treatments. The ANRE and AKRE between N_100_ and N_75_+OICF_25_ were not significant, whereas the ANRE and AKRE for N_50_+OICF_50_ and OICF_100_ were markedly lower than for the N_100_ treatment. Compared with N_100_, N_75_+OICF_25_ and N_50_+OICF_50_ had no significant effect on the APRE. However, OICF_100_ significantly decreased APRE compared with the N_100_ treatment (Fig. 3).

Generally, the effects of the Y and Y × NMS on the NAE, the PAE and the KAE were not significant, but the effects of the NMS on the NAE, the PAE and the KAE were significant (*p* < 0.01). Moreover, except for the effects of Y × NMS on ANRE, the effects of the Y, NMS and Y × NMS on the ANRE, APRE and AKRE were significant (*p* < 0.01) (Table 3).

### Economic benefits

The cost of cultivation, the net return (NR) and the benefit to cost (B:C) ratio were the highest for SRCF+U, whereas they were the lowest for the control. The cost and the B:C ratio for the SRCF+U treatment were slightly higher than for the CF+SCU treatment, whereas the NR for SRCF+U was 9.3% higher than for CF+SCU. The cost for CF+SCU was higher than for N_100_ and N_75_+OICF_25._ However, there was no significant difference in NR and B:C ratio for CF+SCU than for the N_100_. Compared with N_100_, a significantly lower NR was detected for N_50_+OICF_50_ and OICF_100_ with corresponding reductions of 10.8 and 28.9%, respectively. Similarly, the B:C ratio for N_50_+OICF_50_ and OICF_100_ significantly decreased by 12.0 and 29.4%, respectively, compared with the N_100_ treatment (Table 5).

**Table 5:** Economic analysis under the different nitrogen fertilizer management strategies.

Treatment	Seedling raising (yuan ha^−1^)	Chemicals (yuan ha^−1^)	Fertilizer (yuan ha^−1^)	Machine (yuan ha^−1^)	Irrigation (yuan ha^−1^)	Labor (yuan ha^−1^)	Drying (yuan ha^−1^)	Cost of cultivation (Yuan ha^−1^)	^‡^Net return (Yuan ha^−1^)	^‡^Benefit to cost ratio
Control	1,432.5	975	0	3,300	450	4,500	1,500	1,2157.5	2,537.02 e	0.21 c
N_100_	1,432.5	975	2,370	3,300	450	6,000	2,025	1,6552.5	5,059.04 b	0.31 a
N_75_+OICF_25_	1,432.5	975	2,496.8	3,300	450	6,000	2,010	1,6664.3	4,966.82 b	0.30 ab
N_50_+OICF_50_	1,432.5	975	2,623.2	3,300	450	6,000	1,995	1,6775.7	4,332.05 c	0.26 b
OICF_100_	1,432.5	975	3,519.6	3,300	450	4,950	2,055	1,6682.1	3,233.76 d	0.19 c
SRCF+U	1,432.5	975	2,958	3,300	450	6,000	2,295	1,7410.5	5,845.03 a	0.34 a
CF+SCU	1,432.5	975	3,726	3,300	450	5,250	2,085	1,7218.5	5,213.97 b	0.30 ab

**Notes:**

‡ Indicates that the value in the column is the average of 2-year experimental data.

N_100_, 100% conventional nitrogen (N) fertilizer (conventional compound fertilizer and urea); N_75_+OICF_25_, 75% conventional N fertilizer with 25% organic–inorganic compound fertilizer; N_50_+OICF_50_, 50% conventional N fertilizer with 50% organic–inorganic compound fertilizer; OICF_100_, 100% organic–inorganic compound fertilizer; SRCF+U, slow-release compound fertilizer with urea; CF+SCU, compound fertilizer with sulfur-coated urea.

### Comprehensive assessment of the different N fertilizer management strategies

**Figure 4:**
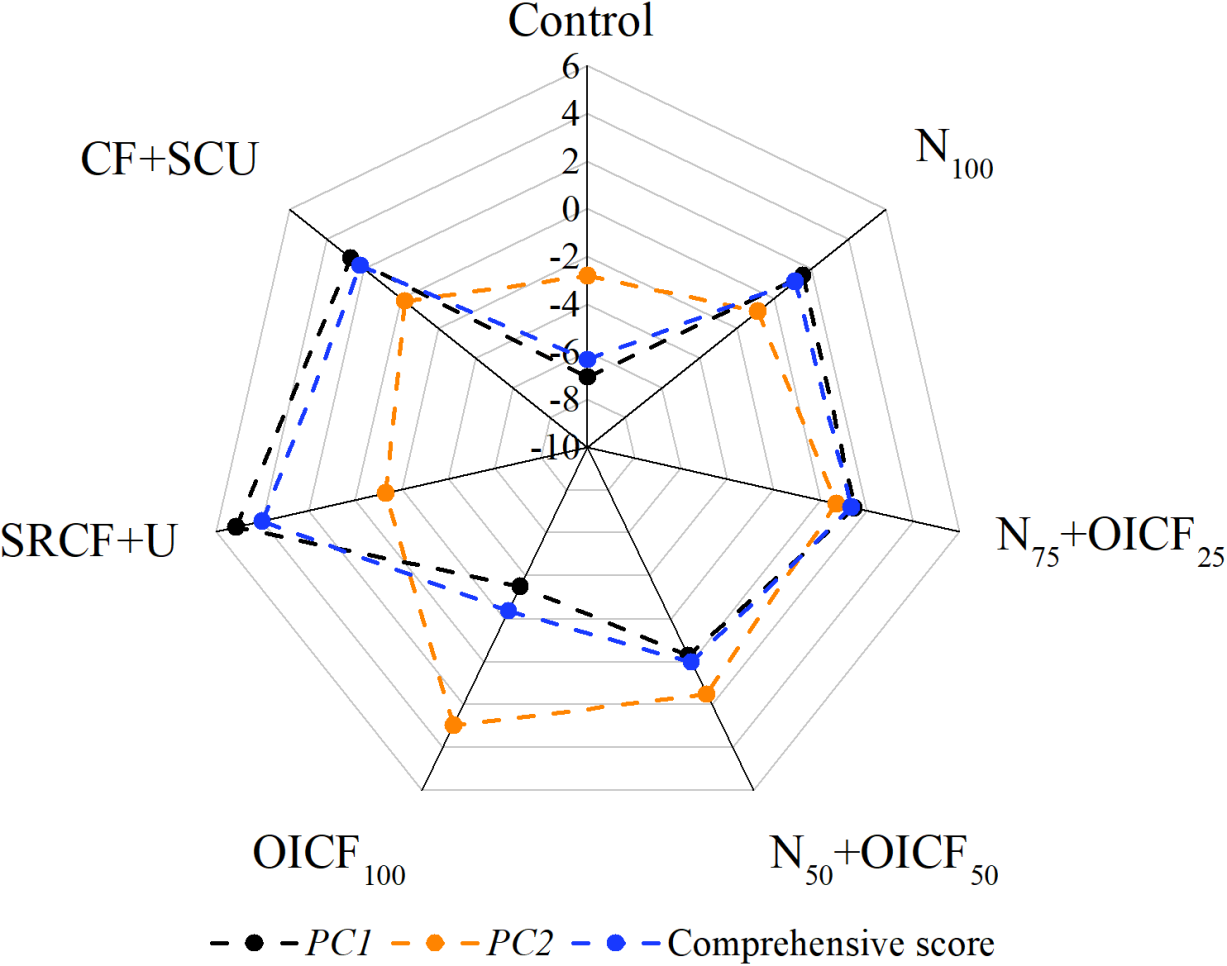
Different principal components score under the different nitrogen fertilizer management strategies. N_100_, 100% conventional nitrogen (N) fertilizer (conventional compound fertilizer and urea); N_75_+OICF_25_, 75% conventional N fertilizer with 25% organic–inorganic compound fertilizer; N_50_+OICF_50_, 50% conventional N fertilizer with 50% organic–inorganic compound fertilizer; OICF_100_, 100% organic–inorganic compound fertilizer; SRCF+U, slow-release compound fertilizer with urea; CF+SCU, compound fertilizer with sulfur-coated urea. Black line, orange line and blue line indicate first principal component score, second principal component score and comprehensive score, respectively.

The principal component analysis was used to estimate the responses of the yield formation, the nutrient uptake and utilization efficiency, and the economic benefits to the different N fertilizer management strategies. The two principal components accounted for 97.4% of the total variance. The first principal component, PC1, explained 80.4% of the total variance, and it was positively correlated with the GY, the TB, the fertilizer uptake related parameters, the fertilizer use efficiency related parameters, and the economic parameters. The second principal component, PC2, accounted for 17.0% of the total variance, and it was positively correlated with the HI, the NHI, the PHI and the KHI. The comprehensive analysis of the rice yield, the nutrient uptake and utilization, and the economic benefits showed that the optimal N fertilizer management was SRCF+U, followed by the CF+SCU treatment (Table 6). The PC1 for SRCF+U and CF+SCU were higher compared with the N_100_, which indicated that the more pronounced effects of SRCF+U and CF+SCU on the GY, nutrient uptake and utilization compared with N_100_. There was insignificant difference between N_100_ and N_75_+OICF_25_. The score of the PC1 and the comprehensive score for N_50_+OICF_50_ and OICF_100_ were lower than for N_100_. However, the score of the PC2 for N_50_+OICF_50_ and OICF_100_ was higher than for N_100_. This indicated that N_50_+OICF_50_ and OICF_100_ negatively affected the grain yield, the nutrient accumulation, and the economic benefits, but were beneficial for the biomass and nutrient translocation to grains (Fig. 4).

**Table 6:** Principal components analysis under different nitrogen fertilizer management strategies.

Statistical parameters	*PC*1	*PC*2
Eigen value	16.9	3.6
% of Variance	80.4	17.0
Cumulative variance (%)	80.4	97.4
Factor loading	Eigen vectors	
Grain yield	0.230	0.169
Total biomass	0.241	−0.037
Harvest index	−0.120	0.453
Grain nitrogen (N) uptake	0.205	0.276
Total N uptake	0.240	0.041
Grain phosphorus (P) uptake	0.212	0.252
Total P uptake	0.242	0.051
Grain potassium (K) uptake	0.223	0.191
Total K uptake	0.240	−0.067
N harvest index	−0.211	0.230
P harvest index	−0.173	0.360
K harvest index	−0.099	0.476
N agronomic efficiency	0.230	0.169
P agronomic efficiency	0.236	0.030
K agronomic efficiency	0.238	−0.004
N recovery efficiency	0.240	0.041
P recovery efficiency	0.230	−0.060
K recovery efficiency	0.237	−0.093
Cost of cultivation	0.202	0.290
Net return	0.242	−0.036
Benefit to cost ratio	0.219	−0.209

## Discussion

### Response of the grain yield to the different N management strategies

Many studies suggested that the combined application of the inorganic fertilizer and the organic fertilizer resulted in a higher crop yield than when each treatment was used alone (Pan et al., 2009; Zhang, Xu & Zhang, 2009; Zhao et al., 2016; Singh et al., 2019). Based on a meta-analysis of 32 long-term experiments in China, Wei et al. (2016) found that the crop yield for the integrated application of inorganic and organic fertilizers was respectively 8% and 29% higher than when the inorganic fertilizer or the organic fertilizer was used alone. In our study, the use of a conventional N fertilizer with an OICF in a 75:25 ratio (N_75_+OICF_25_) or a 50:50 ratio (N_50_+OICF_50_) produced a comparable grain yield than when only the chemical N fertilizer was used. Our results were in accordance with those of Meng et al. (2009), Hidayatullah (2016) and Liu et al. (2017). This was mainly because of the supply of N required during the early growth stage provided by the chemical N fertilizer whereas the organic fertilizer promoted crop growth by supplying N during the later growth stage (Azam Shah et al., 2009). More importantly, the combined application of a chemical N fertilizer with an OICF enhanced the soil nutrient availability, altered the soil microbial community structure, and improved the enzymatic activity and the physical condition of the soil, which promoted root growth and nutrient absorption (Lu et al., 2015; Zhao et al., 2016; Lv et al., 2017). However, a significant reduction in the grain yield was detected when the OICF was used alone compared with the single chemical N fertilizer. The nutrient release rate of the organic fertilizer is slow, which means that using excessive organic fertilizer does not meet the nutritious requirements of the rice plant in its early stage (Zhou, 2012; Liu et al., 2017; Timsina, 2018). In addition, the excessive N supply from the organic fertilizer in the late stage leads to an extended growth and a delayed maturity, which goes against the increase of the rice yield (Xu et al., 2008). This is the principal reason for the lower grain yield of OICF_100_ compared with the N_100_ treatment in the short term. However, the long-term organic fertilization significantly improved the soil fertility and produced a comparably or a higher rice yield than when the chemical fertilizer was used alone (Xu et al., 2008; Wei et al., 2016).

The higher yield indicated the superiority of the treatments using the slow-release fertilizer (SRCF+U and CF+SCU). They produced a higher yield (3.0–8.7%) than when using 100% conventional N fertilizer. This is attributed to higher total biomass at maturity obtained with a slow-release fertilizer than a conventional N fertilizer. A previous study showed that slow-release N fertilizer enhanced the rice yield in comparison with conventional N fertilizer using an alternate wetting and drying irrigation or flooding irrigation (Peng et al., 2014). This is explained by the enhanced N availability in the soil when using the slow-release N fertilizer treatments. This increased the crop N absorption and the accumulation of dry matter in the rice (Peng et al., 2014). However, a lower grain yield of rice was observed when using the slow-release N fertilizer rather than urea in previous researches (Xu et al., 2016; Wei et al., 2017). The nutrient release of the slow-release N fertilizer is significantly impacted by many environmental factors, including the temperature, the soil moisture, and the microbial activity in the soil (Yu et al., 2006). Wei et al. (2017) suggested that the high temperature and humidity conditions during the rice growing seasons in South China prevented the stable release of nutrients form sulfur-coated urea, which reduced the rice grain yield. In addition, nitrification was inhibited by the flooding conditions and the conversion rate of ammonium N to nitrate N decreased, which decreased the nutrient release rate of the slow-release N fertilizer (Xu et al., 2016). Consequently, more experiments on the effects of slow-release N fertilizers on the rice grain yield are required for a wider variety of environmental conditions. In this study, the total biomass significantly increased with the SRCF+U treatment (13.2–13.6%), whereas the harvest index slightly decreased (4.3–5.9%) compared to the N_100_ treatment. The slow-release N fertilizer enhanced the soil N availability, and more N was absorbed by rice plants, resulting in higher total biomass compared with a conventional N fertilizer (Peng et al., 2014). Although the grain yield for the SRCF+U treatment increased significantly (6.6–8.7%), the grain yield increased relatively less than in the total biomass, resulting in a decrease of the harvest index. Similar results were found for rice in an N rich environment (Chen et al., 2014).

Interestingly, the SRCF+U treatment had a higher grain yield than the CF+SCU treatment, which is attributed to a different regime of the N use during the early growth stage. During the early growth stage, the slow-release compound fertilizer was used as basal fertilizer, and urea was used as tillering fertilizer for the SRCF+U treatment. However, the sulfur-coated urea and compound fertilizer were only used as basal fertilizer in the CF+SCU treatment. Wei et al. (2017) showed that the use of slow-release N fertilizer as the basal fertilizer and conventional N fertilizer as the tiller fertilizer increased the accumulation of dry matter, the N absorption, and the rice yield compared with using both the slow-release N fertilizer and the conventional N fertilizer as basal fertilizers.

### N, P and K absorption and utilization efficiency for the different N management strategies

The integrated application of inorganic and organic fertilizers increased the nutrient availability, the microbial and enzymatic activities, the organic matter in the soil, and its physical properties (Wei et al., 2016; Zhao et al., 2016). This improves the biomass production and the nutrient absorption and utilization efficiency (Xu et al., 2008; Abbasi & Tahir, 2012). Compared with N_100_, N_75_+OICF_25_ produced a comparable or a higher N, P and K absorption and utilization efficiency in our study. Similar benefits of combining the chemical N fertilizer with the organic fertilizer have been reported previously (Pan et al., 2009; Liu et al., 2017; Yang et al., 2019). However, OICF_100_ significantly decreased the nutrient use efficiency mainly because of the lower dry matter accumulation and nutrient uptake with a single organic–inorganic fertilizer treatment compared with the 100% conventional N fertilizer treatment. Previous studies reported that adding a large amount of organic fertilizer decreased the N absorption and the panicle number in the early stage of the rice plant, causing a lower N use efficiency and a lower rice yield in the short-term (Zhou, 2012; Liu et al., 2017). However, the long-term application of organic fertilizer significantly enhanced the N uptake and utilization efficiency of rice compared with using a chemical N fertilizer alone (Xu et al., 2008).

In this study, SRCF+U and CF+SCU significantly increased the total nutrient uptake of rice plants compared with the other fertilized treatments. The increased nutrient uptake with slow-release fertilizers are attributed to the larger total biomass because of the significant correlation between the nutrient absorption and the total biomass. In addition, SRCF+U and CF+SCU significantly increased the nutrient utilization efficiency compared with the 100% conventional chemical N fertilizer treatment. A previous study reported that slow-release fertilizers reduced the fertilizer N losses (Xu et al., 2012), which therefore enhanced the nutrient utilization efficiency compared with the conventional N fertilizers (Kiran et al., 2010; Ye et al., 2013; Wang et al., 2015, 2018). Furthermore, the nutrient uptake and utilization efficiency for SRCF+U were higher than for CF+SCU. This indicated that the slow-release compound fertilizer treatment performed better in terms of fertilizer absorption and utilization than the sulfur-coated slow urea treatment. This was mainly attributed to the higher total biomass and nutrient absorption for the SRCF+U treatment compared to the CF+SCU treatment.

The nutrient harvest index for the slow-release fertilizer was lower than for the other fertilized treatments. This indicated that the nutrient is absorbed by the rice plants, but are not efficiently used to improve the grain nutrients. Peng et al. (2014) found that slow-release fertilizers promoted the N uptake in the plant, but decreased the N harvest index of rice. In addition, our results were consistent with a previous study by Zhou (2012), who concluded that the nutrient harvest index increased with the increased substitution ratio of organic fertilizer.

### Economic benefits of the different N management strategies

The cost of cultivation for the slow-release fertilizer treatments was higher than for the 100% conventional N fertilizer treatment. This was mainly because the slow-release fertilizer is more expensive than the conventional fertilizer. The net return (NR) of the slow-release fertilizer treatments was higher than that of the 100% conventional fertilizer treatment. The higher NR was attributed to the higher grain yield obtained for these treatments. A higher NR when using slow-release fertilizers has also been reported previously (Geng et al., 2015). Additionally, another previous study suggested that the NR and the benefit to cost (B:C) ratio for the combined use of organic fertilizer with chemical fertilizer in 50:50 ratio was almost similar to 100% chemical fertilizer (Hasanuzzaman et al., 2010). In our study, a 25% or 50% substitution of the conventional N fertilizer by an OICF resulted in a comparably NR and B:C ratio than with the 100% conventional N fertilizer treatment. The NR and the B:C ratio increased more significantly for the long-term application of the organic and inorganic fertilizers compared with using the inorganic fertilizer alone (Singh et al., 2019). However, the NR and the B:C ratio for a 100% substitution of the conventional N fertilizer by an OICF were very lower than for the 100% conventional fertilizer treatment, which is caused by the lower grain yield for this treatment (Wei et al., 2016). Therefore, the use of an organic fertilizer alone is not profitable for farmers in the short term. However, the long-term single application of the organic fertilizer significantly improved soil fertility which helped to stabilize the productivity and the profitability of the rice plants compared with using a chemical fertilizer alone (Xu et al., 2008; Wei et al., 2016).

### Comprehensive assessment of the different N management strategies

Plotting the different N fertilizer management strategies according to their component scores clearly summarizes the experiments. The first principal component was positively correlated with the grain yield, the total biomass, and the nutrient absorption and utilization efficiency. It separated the plants exposed to the SRCF+U, the CF+SCU, the N_100_ and the N_75_+OICF_25_ treatments from those subjected to the control, the N_50_+OICF_50_ and the OICF_100_ treatments. This indicated that using slow-release fertilizers and a 25% substitution of the chemical N fertilizer by an OICF improved the yield formation and the nutrient uptake and had a higher economic benefit. The second principal component distinguished N_50_+OICF_50_ and OICF_100_ from the other fertilized treatments according to the parameters related to the harvest index and nutrient harvest index. The comprehensive score indeed shows that slow-release fertilizer treatments performed better than the other treatments. In addition, the slow-release compound fertilizer treatment performed better than the sulfur-coated urea treatment. The combined application of the chemical N fertilizer and the OICF in a 75:25 ratio stabilized the yield production, and reduced chemical N added. This was confirmed to be an effective N management strategy for sustainable agricultural production (Abbasi & Tahir, 2012; Wei et al., 2016; Timsina, 2018).

## Conclusion

This study comprehensively evaluated the performances of the different N fertilizer managements on the grain yield, the nutrient absorption and utilization efficiency, and the economic benefits of rice. In this study, slow-release fertilizers enhanced grain yield and nutrient uptake and utilization efficiency and had a higher economic benefit. The slow-release compound fertilizer performed better than the sulfur-coated urea. The combined use of a conventional N fertilizer and an OICF in a 75:25 ratio produced almost similar productivity, nutrient uptake and utilization efficiency as the 100% conventional N fertilizer treatment. This demonstrated that this N management strategy stabilized the yield production and reduced the amount of chemical N used. Our results provide useful information to farmers by outlining their options for the management of N fertilizer in the rice production system.

## Supplemental Information

10.7717/peerj.9596/supp-1 Supplemental Information 1 Raw data.

## Additional Information and Declarations

### Field Study Permissions

The following information was supplied relating to field study approvals (i.e., approving body and any reference numbers): The use of paddy field in which we conduct the experiment was authorized by Jiangsu Zijiang Ecological Agriculture Co., Ltd.

### Data Availability

The following information was supplied regarding data availability: The raw measurements are available as a Supplemental File.

## References

[ref-1] Abbasi MK, Tahir MM (2012). Economizing nitrogen fertilizer in wheat through combinations with organic manures in Kashmir, Pakistan. Agronomy Journal.

[ref-2] Azam Shah S, Mahmood Shah S, Mohammad W, Shafi M, Nawaz H (2009). N uptake and yield of wheat as influenced by integrated use of organic and mineral nitrogen. International Journal of Plant Production.

[ref-3] Bao SD (2000). Analytical methods for soils and agricultural chemicals.

[ref-4] Black CA (1965). Methods of soil analysis part 2.

[ref-5] Chen HF, Feng Y, Cai HM, Xu FS, Zhou W, Liu F, Pang ZM, Li DR (2014). Effect of the interaction of nitrogen and transplanting density on the rice population structure and grain yield in low-yield paddy fields. Journal of Plant Nutrition and Fertilizer Science.

[ref-6] Geng JB, Sun YB, Zhang M, Li CL, Yang YC, Liu ZG (2015). Long-term effects of controlled release urea application on crop yields and soil fertility under rice-oilseed rape rotation system. Field Crops Research.

[ref-7] Guo Z, Liu H, Yuan H, Yang G, Zheng J, Chen L (2015). Insect-proof nets affect paddy field microclimate parameters and grain quality of different japonica rice varieties. Journal of Crop Science and Biotechnology.

[ref-8] Hasanuzzaman M, Ahamed KU, Rahmatullah NM, Akhter N, Nahar K, Rahman ML (2010). Plant growth characters and productivity of wetland (*Oryzasativa* L.) as affected by application of different manures. Emirates Journal of Food and Agriculture.

[ref-9] Hidayatullah A (2016). Influence of organic and inorganic nitrogen on grain yield and yield components of Hybrid rice in northwestern Pakistan. Rice Science.

[ref-10] Iqbal A, He L, Khan A, Wei SQ, Akhtar K, Ali I, Ullah S, Munsif F, Zhao Q, Jiang LG (2019). Organic manure coupled with inorganic fertilizer: An approach for the sustainable production of rice by improving soil properties and nitrogen use efficiency. Agronomy.

[ref-11] Ju XT, Xing GX, Chen XP, Zhang SL, Zhang LJ, Liu XJ, Cui ZL, Yin B, Christie P, Zhu ZL (2009). Reducing environmental risk by improving N management in intensive Chinese agricultural systems. Proceedings of the National Academy of Sciences of the United States of America.

[ref-47] Kiran JK, Khanif YM, Amminuddin H, Anuar AR (2010). Effects of controlled release urea on the yield and nitrogen nutrition of flooded rice. Communications in Soil Science and Plant Analysis.

[ref-12] Li Y, Qiu SF, Zhu RS, Shen JH, Chu YY, Wei GB, Chen G (2015). Effects of organic–inorganic mixed fertilizers on yield formation of machine-transplanted rice. China Soil Fertilizer.

[ref-13] Li Y, Shen JH, Bai JR, Zhu RS, Qiu SF, Zhang W, Wei GB, Xu L, Gao Y (2017). Effects of optimized management of organic–inorganic mixed fertilizers on rice soil fertility. Chinese Journal of Soil Science.

[ref-14] Liu HJ, Chen YW, Sun GF, Chen LG, Zheng JC (2017). Effects of different organic–inorganic fertilizer combination ratios on rice yield and nutrient loss with surface runoff. Chinese Journal of Ecology.

[ref-15] Lu HF, Zhang JW, Yu XC, Zhou HM, Zheng JF, Zhang XH, Liu XY, Chen K, Li LQ, Pan GX (2015). Microbial community diversity and enzyme activity of red paddy soil under long-term combined inorganic-organic fertilization. Journal of Plant Nutrition and Fertilizer.

[ref-16] Lv ZZ, Wu XD, Hou HQ, Ji JH, Liu XM, Liu YR (2017). Effect of different application ratios of chemical and organic fertilizers on soil quality in double cropping paddy fields. Journal of Plant Nutrition and Fertilizer Science.

[ref-17] Meng L, Zhang XL, Jiang XF, Wang QJ, Huang QW, Xu YC, Yang XM, Shen QR (2009). Effects of partial mineral nitrogen substitution by organic fertilizer nitrogen on the yields of rice grains and their proper substitution rate. Scientia Agricultura Sinica.

[ref-18] Mi WH, Sun Y, Xia SQ, Zhao HT, Mi WT, Brookes PC, Liu YL, Wu LH (2018). Effect of inorganic fertilizers with organic amendments on soil chemical properties and rice yield in a low-productivity paddy soil. Geoderma.

[ref-19] Nelson DW, Somers LE (1973). Determination of total nitrogen in plant material. Agronomy Journal.

[ref-20] Ni B, Liu M, Lu S, Xie L, Wang Y (2010). Multifunctional slow-release organic–inorganic compound fertilizer. Journal of Agricultural and Food Chemistry.

[ref-21] Pan G, Zhou P, Li Z, Smith P, Li L, Qiu D, Zhang X, Xu X, Shen S, Chen X (2009). Combined inorganic/organic fertilization enhances N efficiency and increases rice productivity through organic carbon accumulation in a rice paddy from the Tai Lake region, China. Agriculture, Ecosystems & Environment.

[ref-22] Peng S, Buresh RJ, Huang J, Yang J, Zou Y, Zhong X, Wang G, Zhang F (2006). Strategies for overcoming low agronomic nitrogen use efficiency in irrigated rice systems in China. Field Crops Research.

[ref-23] Peng S, Buresh RJ, Huang J, Zhong X, Zou Y, Yang J, Wang G, Liu Y, Hu R, Tang Q, Cui K, Zhang F, Dobermann A (2011). Improving nitrogen fertilization in rice by sitespecific N management: a review. Agronomy for Sustainable Development.

[ref-24] Peng Y, Sun YJ, Jiang MJ, Xu H, Qin J, Yang ZY, Ma J (2014). Effects of water management and slow/controlled release nitrogen fertilizer on biomass and nitrogen accumulation, translocation, and distribution in rice. Journal of Plant Nutrition and Fertilizer Science.

[ref-25] Singh DK, Pandey PC, Nanda G, Gupta S (2019). Long-term effects of inorganic fertilizer and farmyard manure application on productivity, sustainability and profitability of rice-wheat system in Mollisols. Archives of Agronomy and Soil Science.

[ref-26] Soon YK, Kalra YP (1995). A comparison of plant tissue digestion methods for nitrogen and phosphorus analyses. Canadian Journal of Soil Science.

[ref-27] Spiertz JHJ (2010). Nitrogen, sustainable agriculture and food security: a review. Agronomy for Sustainable Development.

[ref-28] Tian HD, Zhang L, Zhang JC, Wang QJ, Xu DB, Halihashi YB, Xu JL, Huang QW (2012). Effect of organic–inorganic compound fertilizers on the growth of rice and wheat in South Jiangsu. Journal of Nanjing Agricultural University.

[ref-29] Timsina J (2018). Can organic sources of nutrients increase crop yields to meet global food demand?. Agronomy.

[ref-30] Walker JM, Barker SA (1962). Absorption of potassium and rubidium from the soil by corn roots. Plant and Soil.

[ref-31] Wang XM, Xie YX, Wang YH, Wang CY, Zhu YJ, Guo TC (2011). Effects of nitrogen application patterns on yields of winter wheat and summer maize and nitrogen use efficiency. Journal of Plant Nutrition and Fertilizer Science.

[ref-32] Wang L, Xue C, Pan X, Chen F, Liu Y (2018). Application of controlled-release urea enhances grain yield and nitrogen use efficiency in irrigated rice in the Yangze River Basin, China. Frontiers in Plant Science.

[ref-33] Wang S, Zhao X, Xing G, Yang Y, Zhang M, Chen H (2015). Improving grain yield and reducing N loss using polymer-coated urea in southeast China. Agronomy for Sustainable Development.

[ref-34] Wei HY, Li HL, Cheng JQ, Zhang HC, Dai QG, Huo ZY, Xu K, Guo BW, Hu YJ, Cui PY (2017). Effects of slow/controlled release fertilizer types and their application regime on yield in rice with different types of panicle. Acta Agronomica Sinica.

[ref-35] Wei WL, Yan Y, Cao J, Christie P, Zhang FS, Fan MS (2016). Effects of combined application of organic amendments and fertilizers on crop yield and soil organic matter: an integrated analysis of long-term experiments. Agriculture, Ecosystems & Environment.

[ref-36] Xu MG, Li DC, Li JM, Qin DZ, Kazuyuki Y, Yasukazu H (2008). Effects of organic manure application combined with chemical fertilizers on nutrients absorption and yield of rice in hunan of China. Scientia Agricultura Sinica.

[ref-37] Xu XJ, Ma HB, Nin YW, Wang JD, Zhang YC (2016). Effects of slow-released nitrogen fertilizers with different application patterns on crop yields and nitrogen fertilizer use efficiency in rice-wheat rotation system. Journal of Plant Nutrition and Fertilizer Science.

[ref-38] Xu J, Peng S, Yang S, Wang W (2012). Ammonia volatilization losses from a rice paddy with different irrigation and nitrogen managements. Agricultural Water Management.

[ref-39] Yang L, Zhou X, Liao YL, Lu YH, Nie J, Cao WD (2019). Co-incorporation of rice straw and green manure benefits rice yield and nutrient uptake. Crop Science.

[ref-40] Ye YS, Liang XQ, Chen YX, Liu J, Gu JT, Guo R, Li L (2013). Alternate wetting and drying irrigation and controlled-release nitrogen fertilizer in late-season rice: effects on dry matter accumulation, yield, water and nitrogen use. Field Crops Research.

[ref-41] Yu LZ, Li DP, Yu SN, Zou JH, Ma T, Wu ZJ (2006). Research advances in slow/controlled release fertilizers. Chinese Journal of Ecology.

[ref-42] Zhang H, Xu M, Zhang F (2009). Long-term effects of manure application on grain yield under different cropping systems and ecological conditions in China. Journal of Agricultural Science.

[ref-43] Zhao J, Ni T, Li J, Lu Q, Fang Z, Huang Q, Zhang R, Li R, Shen B, Shen Q (2016). Effects of organic–inorganic compound fertilizer with reduced chemical fertilizer application on crop yields, soil biological activity and bacterial community structure in a rice–wheat cropping system. Applied Soil Ecology.

[ref-44] Zhao BQ, Yang XD, Li YT, Lin JA, Yan L (2012). Discussions on development of new type fertilizer in China. Phosphate and Compound Fertilizer.

[ref-45] Zheng WK, Zhang M, Liu ZG, Zhou HY, Lu H, Zhang WT, Yang YC, Li CL, Chen BC (2016). Combining controlled-release urea and normal urea to improve the nitrogen use efficiency and yield under wheat-maize double cropping system. Field Crops Research.

[ref-46] Zhou J (2012). Effect of combined application of organic and mineral fertilizers on yield, quality and nitrogen uptake of rice. Journal of Plant Nutrition and Fertilizer Science.

